# An overview of GabRat edge disruption and its new extensions for unbiased quantification of disruptive camouflaging patterns using randomization technique

**DOI:** 10.1371/journal.pone.0300238

**Published:** 2025-07-22

**Authors:** Masahiko Tanahashi, Min-Chen Lin, Chung-Ping Lin

**Affiliations:** 1 Institute of Plant and Microbial Biology, Academia Sinica, Taipei, Taiwan; 2 Department of Life Science, National Taiwan Normal University, Taipei, Taiwan; CONICET: Consejo Nacional de Investigaciones Cientificas y Tecnicas, ARGENTINA

## Abstract

Disruptive colorations are camouflaging patterns that use contrasting colorations to interrupt the continuity of object’s edge and disturb the observer’s visual recognition. The GabRat method has been introduced and widely used to quantify the strength of edge disruption. The original GabRat method requires a composite image where a target object is placed on a particular background. It computes the intensities of ‘frequency components’ parallel and perpendicular to the edge direction at each edge point using Gabor filters, and summarizes the ratios of these two intensities around the perimeter of the shape. However, we found that the original GabRat method has an issue that produces false signals and biases to overestimating the GabRat value depending on the edge angle. Here, we introduce GabRat-R, which can diminish that angle dependency using Gabor filters with randomized base angles. Additionally, we developed GabRat-RR, which iteratively places a target object on a background with random positions and rotation angles to average the effects of the heterogeneity and anisotropy of background. Compared with the original GabRat, our GabRat-R and GabRat-RR programs run more efficiently using multithreading techniques. Those programs are provided as built-in features of the *Natsumushi* 2.0 software and available from the GitHub repository, https://github.com/mtlucanid/GabRat-R.

## Introduction

**Fig 1:**
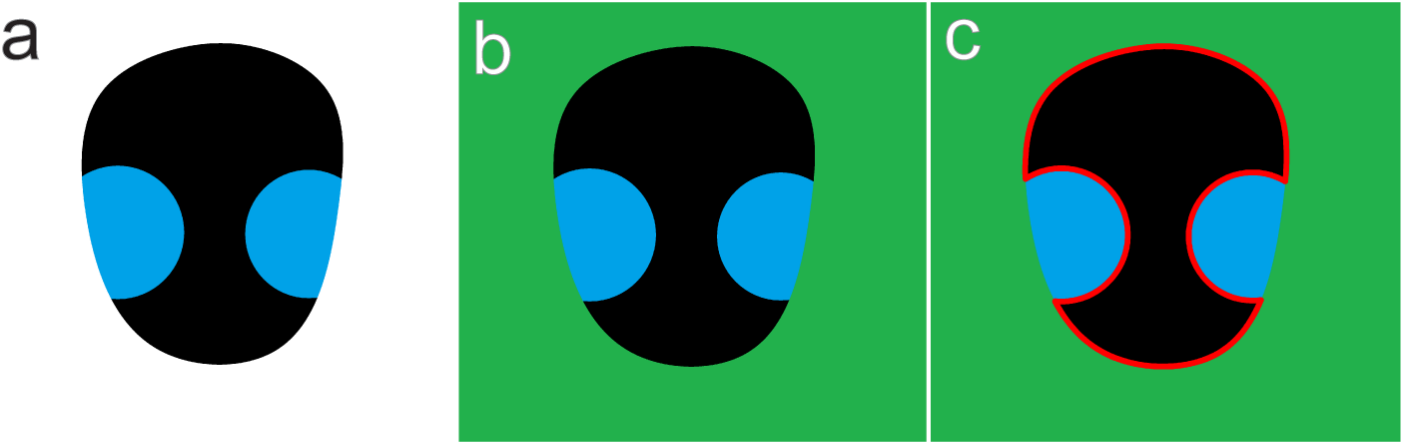
Basic concept of edge disruption effect. (a) A virtual insect image that has black body surface with two blue colorations crossing on the edge. (b) The virtual insect is put on a green background. (c) In this case, visual recognition of the object is disrupted by the blue colorations that create more contrastive false boundaries (red line).

Detection of continuous edges is known to be one of the most fundamental process of visual recognition of target objects in many animals such as humans [1–3], mammals [4], birds [5], fishes [6] and insects [7]. Visual animal predators predominantly use prey’s body outlines to find their prey [8]. To avoid attack, the prey can evolve camouflage to obscure themselves as the first line of defense against the predators [9]. One form of camouflage coloration is the disruptive marginal patterns (edge disruption), in which colors intersect the body edges of the preys. Such colorations are thought to have the effect of disrupting the visual continuity of organisms’ contours and function as camouflage to conceal the actual body boundaries [10]. The effectiveness of edge disruption varies depending on the combination of body coloration and background. Fig 1 illustrates an example of a disruptive camouflage, in which contrasting coloration patterns are in contact with the edge of object’s outline. When the object is put on a white background, the outline of the object is less disruptive because the contrast between the object’s colorations and the background (blue vs. white) is higher than the contrast within the object (blue vs. black) (Fig 1a). However, when the object is on a green background, the contrast between the object and the background (blue and green) becomes lower than the contrast within the object (blue vs. black) (Fig 1b). That produces an illusionary outline (red line), and consequently, the true edge of the object is obscured (Fig 1c). Various methods have been developed to quantify the degree of edge disruption of an object [11–16]. Some quantitative methods use simple mathematical operations, such as Gabor filters, to provide relatively objective and general-purpose assessment of edge disruption [16]. On the other hand, other methods rely heavily on computer vision techniques such as Canny edge detector [11] and SIFT feature detection [16], which ultimately require thresholding that is sensitive to artificially defined parameters. In this study, we first introduce the GabRat edge disruption method, originally developed by Troscianko *et al*. [16], which uses Gabor filters as its main mathematical operation. Secondly, we identify several issues associated with the calculation of GabRat. Finally, two new methods, namely, GabRat-R and GabRat-RR, are proposed to solve those issues and extend the application of the original GabRat.

## Overview of basic GabRat calculation

**Fig 2:**
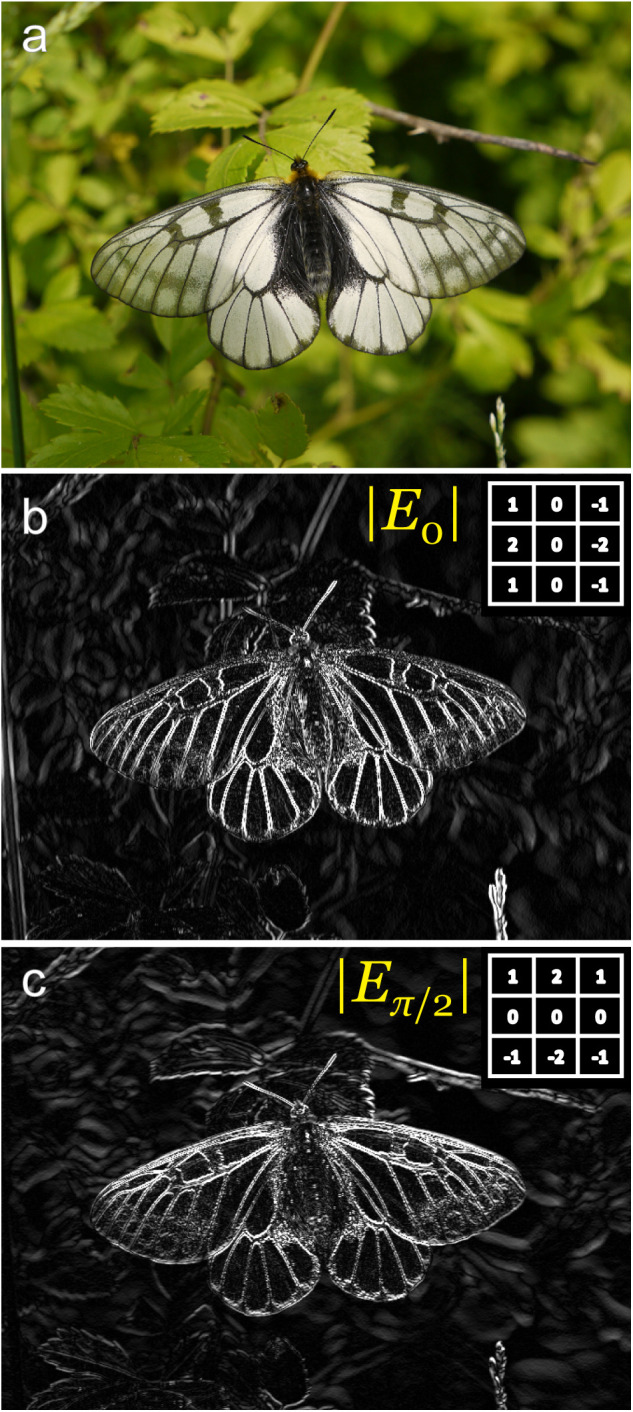
Examples of 3 × 3 Sobel filter matrices and their outputs. Sobel filters can be considered the simplest case of Gabor filters. (a) A sample photograph of the glacial Apollo butterfly, *Parnassius glacialis*. (b) Absolute vertical edge energy (*θ* = 0) and (c) absolute horizontal edge energy (*θ* = π/2).


GabRat is a method designed to quantify the effect of edge disruption when a target object is placed on a specific background image [16]. The strength of edge disruption can be defined as the relative intensity of contrastive local patterns perpendicular to the edge of the object, because such patterns often disturb the visual recognition of the true object’s outlines. GabRat uses Gabor filters [17], which are generally used to determine the intensity of spatial frequency components in specific directions. Fig 2 shows examples of extracting spatial frequency components in vertical and horizontal directions using 3 × 3 Sobel filters, which can be considered the simplest Gabor filters. Before the filter operation, a digital color image (Fig 2a) is converted to a monochrome image or separated into RGB channels, so that each pixel of the image has a single intensity (brightness) value of 0.0 to 1.0. To calculate the vertical energy at a specific pixel (= *E*_*i*, *j*_), the intensity of an upper-left neighboring pixel (= *P*_*i*–1, *j*–1_) is multiplied by the value of the filter matrix at the corresponding location (= 1), the upper neighboring pixel (= *P*_*i*, *j*–1_) is multiplied by zero, the same applies hereafter, and the results are summed up as follows:

$$\begin{array}{c} E_{i,\;j}=\;P_{i-1,\;j-1}\;-\;P_{i+1,\;j-1}\;+\;{2P}_{i-1,\;j}\;-\;{2P}_{i+1,\;j}\;+\;P_{i-1,\;j+1}\;-P_{i+1,\;j-1} \end{array}$$

Since the positives and negatives of the filter matrix elements are reversed across the vertical axis, perpendicular patterns (e.g., horizontal zebra patterns) are canceled in the matrix calculation. As a result, the vertical Gabor filter selectively passes the spatial frequency contents with vertical patterns (Fig 2b). Similarly, horizontal energy of the image at each location can be calculated using the horizontal Gabor filter (Fig 2c).

GabRat calculation uses a set of Gabor kernel filters that are determined by four parameters: kernel size (*σ*), aspect ratio (*γ*), frequency (*Fx*) and number of filter angles (*nAngles*). Of those, *σ* needs to be determined for each analysis, depending on the pixel size of the target object, so that the kernel filter sufficiently covers the edge boundary and its surrounding area (the size of the filter matrix, *k × k*, is determined as *k = *6*σ* + 1). The aspect ratio *γ* is basically fixed to 1.0. Changes of *Fx* alter the frequency of the zebra pattern of Gabor filter (Fig A in S1 Appenidx); however, it is not recommended to use a large *Fx* value (> 2.0) because the result becomes overly sensitive to the specific recurrent pattern with that frequency. In this study, we only tested *Fx *= 2.0, following the previous studies [16,18].

**Fig 3:**
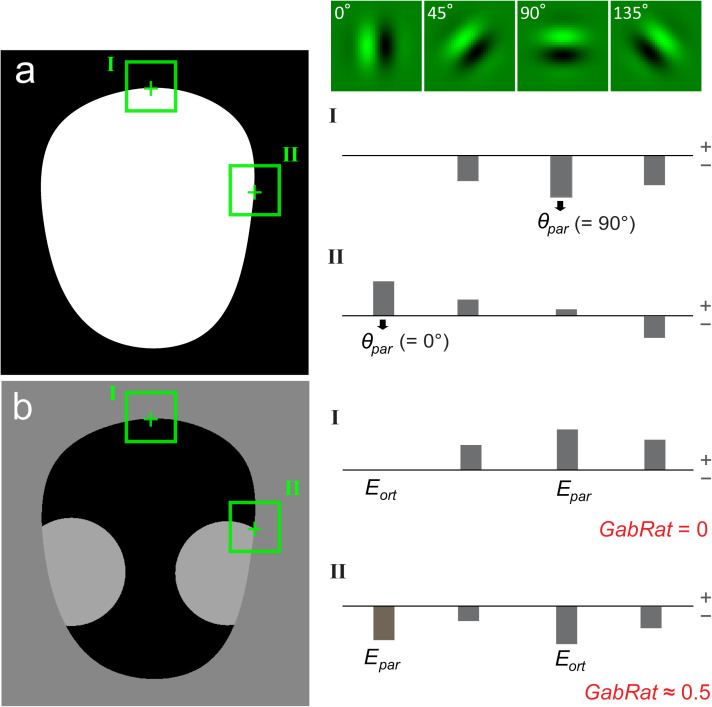
The principle of GabRat calculation. The model image is same as Fig 1a. (a) At the first step, edge energies of four different angles (*nAngles = 4*) are calculated on a binary (black and white) image along the object edge using Gabor filters. The filter angle that gives the maximum absolute edge energy (*θ*_*par*_) is referred to as the ‘edge angle’ at each location. (b) Next, edge energies of four different angles are calculated on a grayscale image in the same way. Of those, edge energy at the angle of *θ*_*par*_ is chosen to be the parallel energy (*E*_*par*_). Orthogonal energy (*E*_*ort*_) is simply defined as the edge energy that is perpendicular to *θ*_*par*_. A local *GabRat* value is calculated as *GabRat* = |*E*_*par*_|/ (|*E*_*par*_| + |*E*_*ort*_|) on each point.

The *nAngles* parameter is the essence of GabRat calculation, and also the root of all issues identified in the next section. In GabRat calculation, typically, four Gabor kernel filters of different rotation angles (i.e., 0, *π*/4, *π*/2 and 3*π*/4 when *nAngles* = 4) (Fig A in S1 Appendix) have been used to determine ‘edge energy’ of specific direction around each edge point [16,18]. GabRat first determines the ‘edge angle’ on each pixel that constitutes the edge outline. To do this, it temporally generates a binary (black-and-white) image, in which black (= 0) represents the background and white (= 1) represents the area of the foreground object (Fig 3a). GabRat picks every edge pixel and calculates the edge energies of four different directions on the binary image using Gabor kernel filters corresponding to those angles. Hereafter, the angle at which the absolute energy (i.e., absolute value of the edge energy) becomes highest is called the parallel angle (*θ*_*par*_). It should be noticed that in most locations *θ*_*par*_ is not exactly parallel with the tangential line of the true edge. In other words, the continuous edge angle is discretized into four angles (0, π/4, π/2 and 3π/4) by this operation. The orthogonal angle (*θ*_*ort*_) is simply assigned to be perpendicular to *θ*_*par*_. Next, the same GabRat filters are applied to the real (grayscale) image in order to calculate four absolute energies |*E*_0_|, |*E*_*π*/4_|, |*E*_*π*/2_| and |*E*_3*π*/4_| at the corresponding edge point (Fig 3b). Of those, the parallel energy |*E*_*par*_| and orthogonal energy |*E*_*ort*_| are chosen according to the determined edge angles *θ*_*par*_ and *θ*_*ort*_, respectively. Notice that when the real image has some disruptive patterns at that location, |*E*_*par*_| does not always indicate the maximum value of the four absolute energies (Fig 3b, position II). Finally, GabRat value on that edge point is defined as: *GabRat* = |*E*_*ort*_|/ (|*E*_*par*_| + |*E*_*ort*_|). According to this definition, GabRat value exceeding 0.5 means that the orthogonal energy is larger than the parallel energy at that specific location, therefore indicates that the false edge is more contrastive than the true edge. In most analysis, GabRat values are summed up for all edge pixels on the object’s contour and the mean GabRat value is used to represent the level of edge disruption of the target object against the specific background [16].

The original GabRat was implemented as a plugin for the micaToolbox [15] of the ImageJ software [19,20] and its Java source code is available under the GPL-3.0 license in the public depository GitHub (https://github.com/troscianko/micaToolbox).

## Issues in GabRat calculation

**Fig 4:**
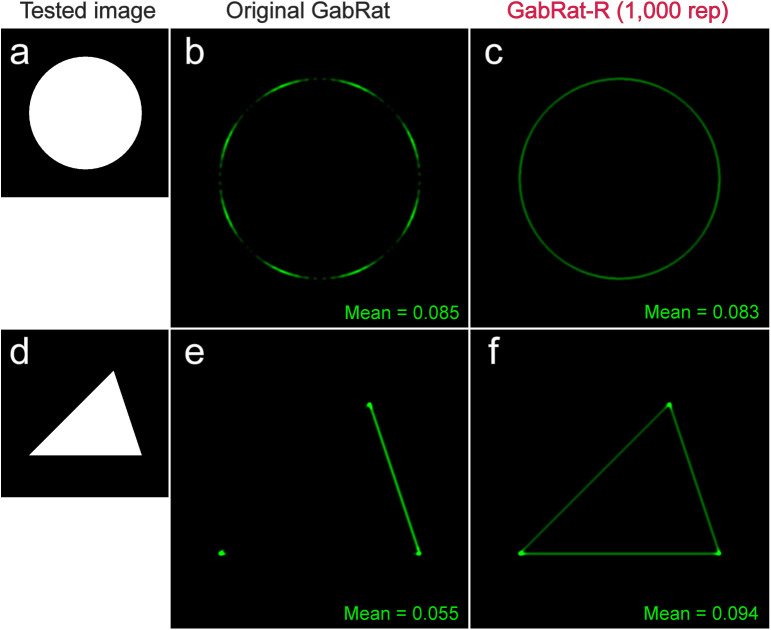
Comparison of the original GabRat and GabRat-R. (a) A tested image. This is the simplest pattern, in which a white circular object (200 pixels in radius) is placed on the black background. Since there is apparently no ‘false-edge’ in this picture, the edge disruption effect is expected to be constant and nearly zero everywhere on the edge points. (b-c) Results of GabRat and GabRat-R (1,000 iterations), respectively. Intensity of the green signals represents GabRat values ranging from 0.0 to 1.0 at each location. An angle-dependent, periodic pattern is seen in the original GabRat. (d-f) Similar comparison using a triangular shape. *σ* = 6.0, *γ* = 1.0, *Fx* = 2.0, *nAngles* = 4, *baseAngle* = 0.

**Fig 5:**
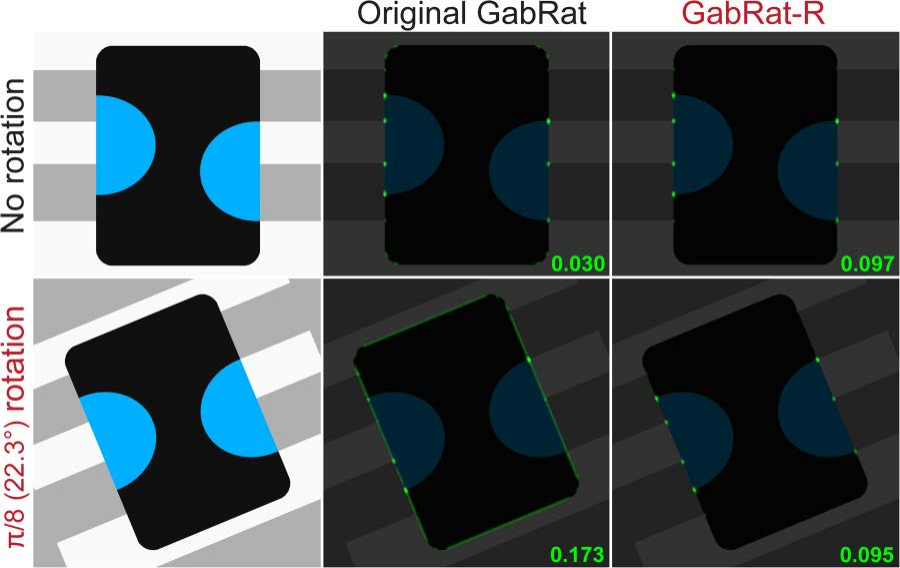
Rotation-dependent false signals in the original GabRat and averaged false signals in GabRat-R. *GabRat* values at each location are presented as the green signals superimposed on the original images. The mean values are shown at the bottom-left of the panels.

Although GabRat value is a useful index to quantify the effect of edge disruption, we noticed that the original GabRat calculation has an intrinsic issue, which causes the angle-dependent false signal pattern. Fig 4 shows this false signal pattern using the simplest foreground and background models. When a white circular object is put on a black background (Fig 4a), the strength of edge disruption is expected to be zero on every edge point, since the object has no apparent false edge. However, the original GabRat calculation (*σ* = 6, *nAngles* = 4) exhibits an angle-dependent periodic signal pattern, the intensity of which ranges from 0.000 to 0.188 (mean = 0.085) (Fig 4b and Table 1). This type of false signal pattern is more pronounced with shapes that include straight lines, especially when the angle of the straight line is distant from a multiple of *π*/4 (Fig 4d,e). Moreover, the intensity of the false signal is dependent on the rotation of the entire image. In Fig 5, we first prepared a composite image in which an insect model with edge disruptive patterns was placed on a background, then rotated the entire image by *π*/8 (= 22.5°). Although those two composite images were identical except for their placement within the computer screen, the mean GabRat values varied from 0.030 to 0.173, which is mainly caused by the false signals on the edges with straight lines (Fig 5).

**Table 1:** Effect of the number of Gabor filters (*nAngles*) on GabRat calculation. Target object is a white circle (200 pixels in radius) on the black background. *σ* = 6.0.

*nAngles*	GabRat-Min	GabRat-Max	GabRat-Mean
2	0.0000	0.4871	0.1918
4	0.0000	0.1882	0.0847
8	0.0000	0.0829	0.0405
16	0.0000	0.0619	0.0218
32	0.0000	0.0485	0.0197
64	0.0000	0.0462	0.0124

**Fig 6:**
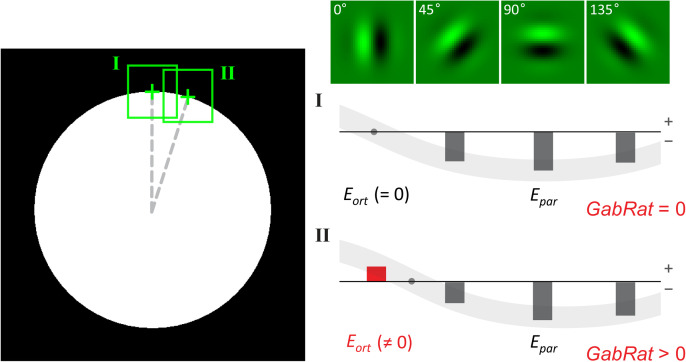
The cause of the angle-dependent false signals in the original GabRat calculation. The model image and GabRat parameters are the same as Fig 5a. Since the binary image is identical to the real image, *E*_*k*_ is always equal to *E’k*. At the upper-most position of the circle edge (I), *E*_*par*_ takes a large negative value (because the black-and-white pattern of the local area opposes the kernel filter pattern) whereas *E*_*ort*_ = 0, thus, *GabRat* = 0. However, at the second position (II), *E*_*ort*_ takes a small negative value due to the discrepancy between actual tangential angle and the kernel filter angle. As a result, *GabRat* takes a nonzero value.

Why does such a false signal occur in GabRat calculation? The answer is because there is a mismatch between a discrete edge angle (*θ*_*par*_) determined by the limited number (*nAngles*) of Gabor filters and the actual angle of the edge outline, as mentioned in the previous section. Fig 6 shows the same circular model image as Fig 4a, in which the binary image (i.e., a temporal image that GabRat program automatically generates, see Fig 3a) is identical to the real image. At the upper-most position of the circle (Fig 6, position I), the parallel angle *θ*_*par*_ = 0 and *E*_*par*_ takes a large negative value (since the kernel filter pattern opposes the black-and-white pattern of the local image) whereas *θ*_*ort*_ = *π*/2 and *E*_*ort*_ = 0 (as the kernel filter pattern is perpendicular to the local image pattern); therefore, *GabRat* = |*E*_*ort*_|/ (|*E*_*par*_| + |*E*_*ort*_|) = 0. However, at the second position (Fig 6, position II), *θ*_*par*_ and *θ*_*ort*_ remain unchanged (*θ*_*par*_ = 0, *θ*_*ort*_ = *π*/2) but *E*_*ort*_ takes a nonzero value; therefore, *GabRat* > 0. The easiest solution to reduce the false signal is to increase the *nAngles* parameter, since *θ*_*par*_ becomes closer to the actual edge angle (Table 1). However, increasing *nAngles* parameter significantly decreases the mean GabRat values (Table 1), therefore, GabRat values calculated with different *nAngles* parameters cannot be directly compared with those determined in the previous studies, which typically use *nAngles* = 4.

## GabRat-R

Here we developed an improved method, namely GabRat-R, which can almost flatten the false signal but still keeps a sufficient mean-level compatibility with the original GabRat method. GabRat-R essentially uses the same number of Gabor filters (typically, *nAngles* = 4) to determine *θ*_*par*_ as used in the original GabRat. Further, GabRat-R internally uses an additional *baseAngle* parameter, which represents an offset rotation angle of each Gabor filter (Fig A in S1 Appendix). The GabRat-R repeatedly calculates the GabRat values (e.g., 1,000 repetitions) with a randomly generated *baseAngle* parameter (0 ≤ *baseAngle* < *π*) based on the Mersenne twister algorithm [21] for each iteration. The number of iterations is specified by the *nRepeat* parameter, which is typically set to 1,000. The GabRat-R method is provided as built-in features of the *Natsumushi* 2.0 image analysis software [22,23].

**Fig 7:**
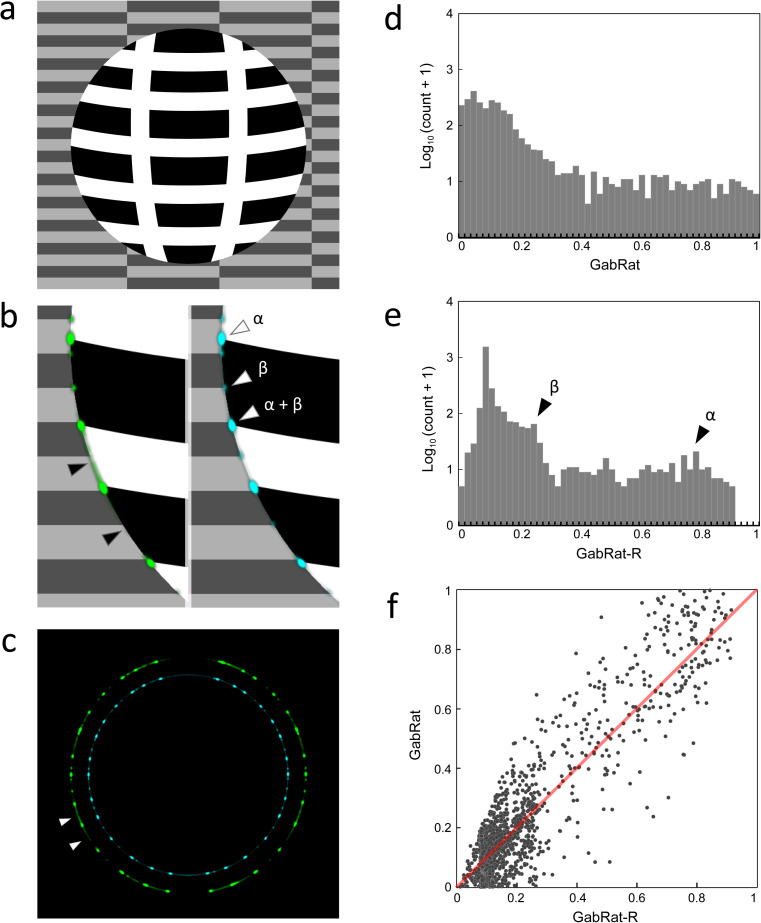
Difference of spatial structures of GabRat signals in the original GabRat and GabRat-R. (a) A model object put on a background image. The model object has stripe patterns with high contrast whereas the background consists of block-like patterns with low contrast. (b, c) Difference in fine signal distributions between GabRat (green) and GabRat-R (blue). Arrowheads indicate (α) the location where high contrast patterns on the object intersects the edge outline and (β) the location where low contrast patterns on the background intersects the edge outline. (d, e) Frequency distributions of the signal intensity of GabRat and GabRat-R, respectively. (f) Correlation between GabRat and GabRat-R. A dot represents the intensities of GabRat and GabRat-R at a specific pixel on the edge outline. A red line indicates the equilibrium line (*y* = *x*).

In the cases of Fig 4, GabRat-R nearly flattened the false signal along the entire edge outline of the object, but the mean GabRat values were still close to that of the original GabRat method (Fig 4c,f). Another advantage of GabRat-R over GabRat is that the flattened false signal makes the spatial structure of the edge disruption intensity more obvious. Fig 7 illustrates the difference in the spatial structures of GabRat vales between the original GabRat and GabRat-R. In this verification experiment, a circular white model object with disruptive black stripes was put on a background image with grey tiled patterns (Fig 7a). There were four types of edge points in terms of disruptive patterns: α) the points where a boundary of the stripe pattern of the object (black & white) intersects with the edge outline of the object, β) the points where a boundary of the stripe pattern of the background (dark grey & grey) intersects the edge outline of the object, α + β) both α and β happen coincidently, and γ) the other locations where no disruptive pattern exists. Since the contrast of the object’s coloration was higher than that of the background pattern, it was expected that stronger GabRat signals would appear at α locations and weaker signals β locations. In the original GabRat calculation, some β signals almost disappeared due to the interference of background noise, whereas those local GabRat peaks were clearly seen in the result of GabRat-R (Fig 7b,c). These α and β peaks were also clearer in the histogram of GabRat values in GabRat-R calculation (Fig 7d,e). The difference in signal intensities between the original GabRat and GabRat-R at the same location tended to be larger when the GabRat value shows intermediate edge disruption (*GabRat* ≈ 0.5) (Fig 7f).

In addition to the new randomized *baseAngle* feature, the algorithm of GabRat itself has also been improved to significantly increase the calculation speed, which is important for the iterative calculation of GabRat in the GabRat-R method. Our new GabRat program is written in C++ and runs for native CPUs, giving it a speed advantage over the original GabRat program, which is provided as the micaToolbox plugin function [15] in the Image J software [19,20] and runs in the Java virtual machine. Furthermore, GabRat-R uses multithreading techniques to perform each iterative GabRat calculation in parallel, further improving its computation speed. First, to evaluate the efficiency of pure algorithms regardless of the execution environment, we converted the original GabRat Java program into a C++ program mostly as it was (in a word-by-word manner, since these two computer languages have quite similar syntax), and composed it into a single C++ function. For the detailed process of conversion, see the supporting information (S1 Appendix). After that, two C++ source codes of the original GabRat and our new GabRat program were compiled in Visual C++ 2005 (Microsoft) with the moderate optimization for execution speed (/O2). As a test image, we prepared a composite image of a flightless Easter Egg weevil, *Pachyrhynchus tobafolius* (Coleoptera: Curculionidae), placed on a natural background (Fig B in S1 Appendix). We measured the computational speed of the two programs by running them for a single iteration and 1,000 iterations with different *σ* values (4.0 and 8.0). As a result, the improved GabRat program achieved approximately three times speedup compared to the original GabRat (Table 2). Next, we evaluated the multithreading performance of the GabRat-R program, which requires repeated calculations of GabRat. GabRat-R was designed to execute the improved GabRat algorithm in parallel, further increasing the computation speed significantly if the CPU supports the multi-threading technology (Table 2).

**Table 2:** Comparison of the calculation time among the original GabRat implementation (rewritten in C++), the improved algorithm of our GabRat, and the GabRat-R which computes the improved GabRat in parallel. *nAngles* = 4.

*σ*	Iteration	Original GabRat	Improved GabRat	GabRat-R					
				1 thread	2 threads	4 threads	6 threads*	8 threads	12 threads
4	1	46 ms	16 ms	–	–	–	–	–	–
	1,000	39.1 s	15.6 s	12.2 s	6.3 s	3.2 s	2.2 s	2.5 s	2.3 s
8	1	125 ms	47 ms	–	–	–	–	–	–
	1,000	124.7 s	49.5 s	45.7 s	23.0 s	11.9 s	8.8 s	9.5 s	8.5 s

* Default number of threads in the tested environment (Intel Core i7-990X, 6 cores/12 threads, overclocked to 4.5 GHz). The GabRat-R program automatically set it to the number of CPU cores.

## GabRat-RR

The original GabRat method first requires a composite image where a target object is placed on a particular background image (e.g., Fig 7a). Since GabRat calculates edge energies by looking at patterns of both the object and the background near its edges, the background fundamentally influences the GabRat value (e.g., Fig 7b). Natural backgrounds, such as tree leaves and tree trunks, usually consist of different local patterns and often have angle-dependent components. Therefore, intensity of the edge-disrupting effect may also depend on the position and the angle where the foreground object is placed.

**Fig 8:**
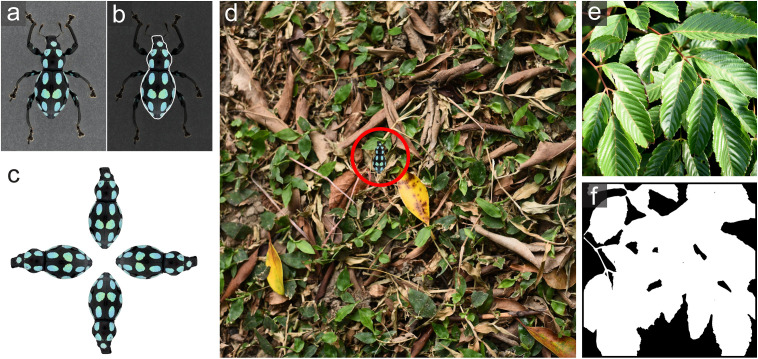
An analytical procedure of GabRat-RR. (a) An original insect image. (b) A region of interest (ROI) on the insect image. (c) Four rotated insect images by π/2 step. (d) GabRat-RR randomly selects one of the rotated ROIs, puts it at the random position on the background, and calculates a *GabRat* value by using a randomly generated *baseAngle* parameter. This procedure is repeated *nIndividual* times. (e) Another background image. (f) A mask image to specify the area where the object is allowed to be placed.

GabRat-RR method was thereby developed to average the effects of the heterogeneity and anisotropy of background images. The GabRat-RR method is also provided as built-in features of the *Natsumushi* 2.0 image analysis software [22,23]. Prior to the GabRat-RR computation, a foreground image (i.e., a picture containing a target object) (Fig 8a) and a background image must be prepared separately. To specify the range of the target object (e.g., the body part of a weevil excluding antennae and legs), a region-of-interest (ROI) must be specified on the foreground image (Fig 8b). GabRat-RR repeatedly generates composite images of the foreground object placed onto the background image, using random positions and rotation angles (Fig 8c,d) and calculates the GabRat values. In each iteration, GabRat-RR also generates a random *baseAngle* parameter (as mentioned in GabRat-R) to rotate the Gabor filters and eliminate the angle-dependent false signals. This dual-randomization feature is the origin of the name GabRat-RR. The number of the iteration is specified by *nIndividual* parameter. As a result, each location on the object’s contour has *nIndividual* distinct GabRat values because the adjacent background patterns are different. At the end of the iterated computation, GabRat-RR summarizes the results in two ways: 1) list of the mean GabRat values for each iteration, in which each value represents the intensity of edge disruption of an entire shape, and 2) means of the GabRat values at each location of the object’s contour. The first summary is related to the heterogeneity and anisotropy of the background, and the second summary shows the spatial distribution of GabRat intensity on the object.

The rotation angle of the target object is currently restricted to four discrete values: 0, 45°, 90°, and 135° (Fig 8c). That is because other intermediate rotations always cause pixel-level rearrangement of the object’s contour and this changes the number of edge pixels, making it difficult to summarize the results in the second form. We have already found a technical solution that allows the use of continuous random rotation angles, and this improvement will be applied in future versions of the GabRat-RR program.

Natural background images often contain areas which are not suitable for the target object to be placed. For example, a flightless *Pachyrhynchus* weevil cannot stay in the empty space between leaves (Fig 8e). In such cases, GabRat-RR can use an optional mask (black and white) image to specify the valid areas of background (Fig 8f). If a mask image is present, GabRat-RR program will place the target object only within the valid areas defined by white pixels in the mask image.

## Comparison of original GabRat, GabRat-R and GabRat-RR

**Fig 9:**
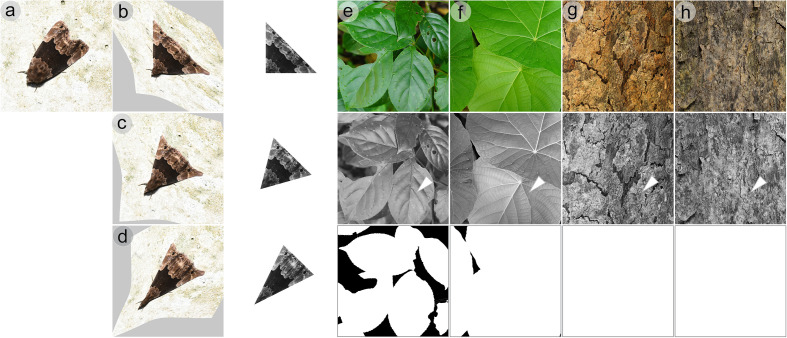
A natural model images and natural backgrounds used for the comparison of the original GabRat, GabRat-R and GabRat-RR. (a) An image of the moth *Dinumma deponens*. (b-d) To simulate the experimental conditions of previous studies, the moth image was deformed into three different triangular shapes using TPS transformation. The deformed images were then cut into accurate triangles, resized so that they all have the same area size, and converted into grayscale images. (e-h) Original background photographs (top) and grayscale images (middle). Arrowheads indicates the fixed location where the triangular moth images were put in GabRat-R analysis. To specify the valid area within which the moth images can be placed in GabRat-RR, the actual areas of leaves and tree trunks were painted white and the gaps between the leaves were painted black (bottom).

This study demonstrated that GabRat value from the original GabRat method is significantly affected by the angles of edge outline, especially when it includes straight lines (Figs 4 and 5). Most previous studies used isosceles triangles as template shapes, mimicking a moth perched on an environmental background [24,25]. In such studies, false GabRat signals might possibly have happened depending on two factors: 1) the shape of the triangle, and 2) the direction of head. Here, we aimed to verify the existence of biased GabRat evaluation in those common experimental cases. For a model organism, we choose a moth species, *Dinumma deponens*, which is common in Taiwan and has typical disruptive colorations on the wing (Fig 9a). A living specimen of *D. deponens* was photographed in Ma-Mei forest trail, Hsinchu County, Taiwan (Fig 9a), and the outlines of the moth was transformed into isosceles triangular shapes with vertex angles of 90° (right triangle: Fig 9b), 60° (equilateral triangle: Fig 9c) and 45° (acute triangle: Fig 9d) by the thin plate spline (TPS) deformation using *Natsumushi* 2.0 software [22,23]. The deformed images were trimmed by exact isosceles triangles, resized so that the area of the triangles become approximately 10,000 pixels, and converted into grayscale images (Fig 9b–d). For background images, two pictures of the leaves of *Bischofia javanica* and *Macaranga tanarius* trees and two pictures of the trunks of *B. javanica* in different appearances were resized into 3,000 × 3,000 pixels so that the length of each side corresponds to 15 cm in the real objects, and finally converted into grayscale images (Fig 9e–h).

**Fig 10:**
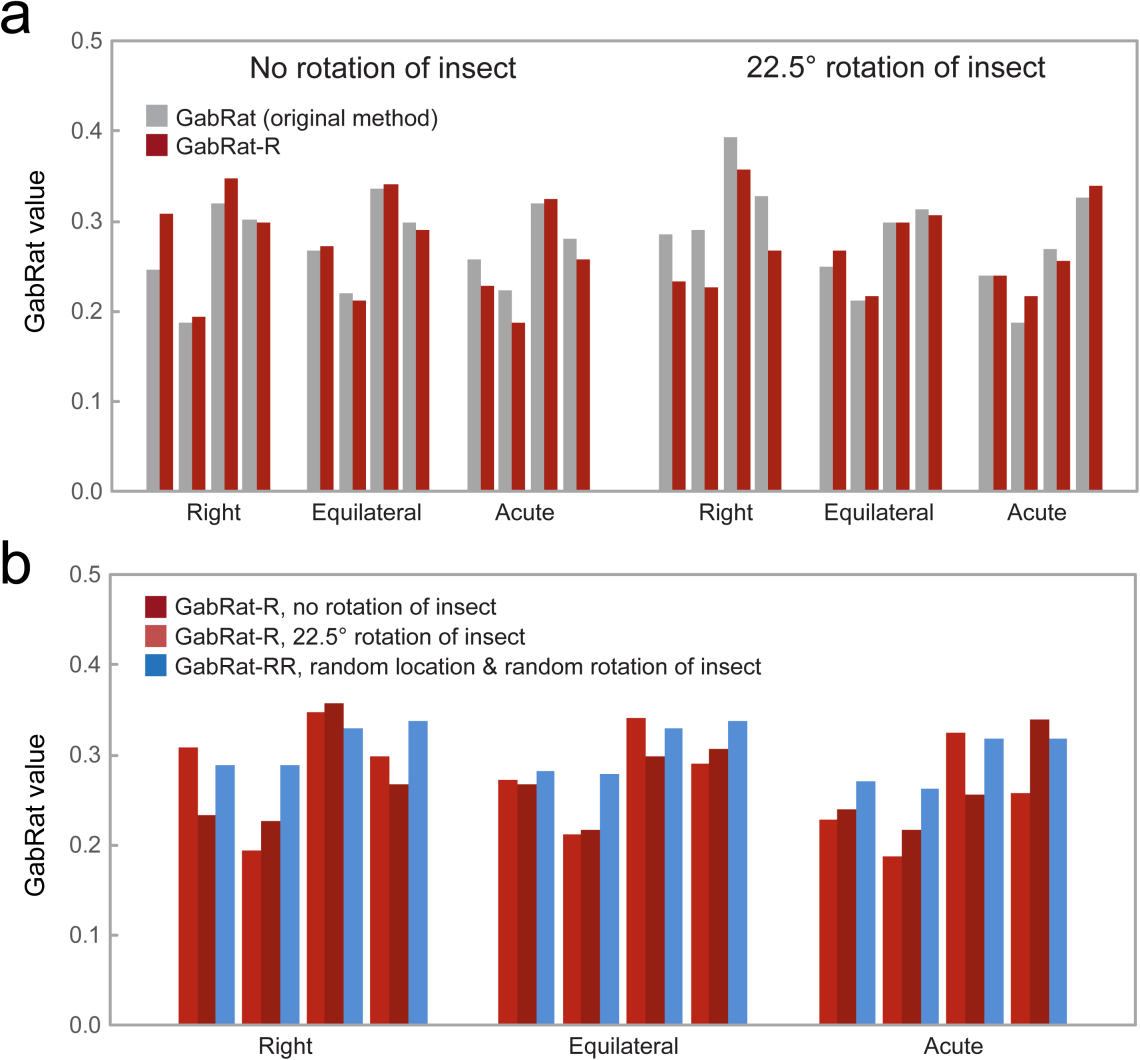
Comparison of different GabRat methods. (a) Comparison of the original GabRat and GabRat-R, using the moth images of three triangular shapes putting at the fixed position of the four different natural backgrounds (see Fig 9). (b) Comparison of GabRat-R (fixed position and rotation angle) and GabRat-RR (randomized position and rotation angle, *nIndividual* = 1,000). *σ* = 6.0.

To test the difference in original GabRat and the GabRat-R, those three triangular moth images were put on a fixed position of each background pictures (arrowheads in Fig 9e–h) with no rotation (0°) and π/8 (22.5°) rotation. For the original GabRat, the ‘random base angle’ function was disabled and *nRepeat* = 0 (that enforces the application to use the original GabRat method), whereas GabRat-R uses the ‘random base angle’ function with *nRepeat* = 1,000. The result showed that the difference in GabRat-R and GabRat-RR tend to be greater when using a right triangle shape, especially when the moth images were rotated by 22.5° (Fig 10a). Additionally, we also tested GabRat-RR to clarify the effect of heterogeneity in background. Since the pictures of the tree leaves contain some empty areas where a moth cannot settle, we use the mask images to specify the valid region (Fig 9e,f). When the results of GabRat-RR were compared with those of GabRat-R at two rotations, substantial differences were found within each combination of moth shapes and backgrounds (Fig 10b). However, no clear tendency was observed between those two analyses, possibly due to the low heterogeneity of the background images.

## Conclusion and applications

The original GabRat calculation has an intrinsic issue that generates false signals and they may affect the mean *GabRat* value in certain cases, especially when the target shapes have long, straight edge outlines with intermediate angles. GabRat-R averages out those angle-dependent issues, allowing unbiased comparisons among different shapes. Additionally, GabRat-RR helps account for the heterogeneity and anisotropy in background images. The robust and unbiased quantification of edge disruption intensity provided by GabRat-R/RR can be considered as an objective feature index of coloration and can be useful in combination with other indices to describe the coloration of an object (e.g., [26]).

## Supporting information

S1 Appendix Supplementary text, figures and codes. (DOCX)
