# Supplementary material for: An overview of GabRat edge disruption and its new extensions for unbiased quantification of disruptive camouflaging patterns using randomization technique
Source: PLoS One. 2025 Jul 22;20(7):e0300238. doi: 10.1371/journal.pone.0300238 (PMC12282856; doi:10.1371/journal.pone.0300238)
Supplement: S1 Appendix — (DOCX) [file pone.0300238.s001.docx]

Supporting information for

An overview of GabRat edge disruption and its new extensions for unbiased quantification of disruptive camouflaging patterns using randomization technique

Masahiko Tanahashi, Min-Chen Lin, Chung-Ping Lin

**Materials and methods**

*Conversion of original GabRat program*

The original GabRat is available as a plugin function of mica Toolbox (Troscianko & Stevens, 2015) of ImageJ software. The original Java source code is available under the GPL-3.0 license on the public code depository GitHub (https://github.com/troscianko/micaToolbox). To evaluate the efficiency of algorithm besides the difference in the running environment, we converted the Java program of original GabRat into C++ program mostly as it was, and composed it into a single C++ function (Codes A and B). In this conversion process, Math library calls in Java (e.g. Math.sin) were replaced with the corresponding standard C++ library calls. Declarations of new arrays (e.g. int[] foo = new int[n]) were substituted by the equivalent C++ pointer allocations (e.g. int* foo = new int[n]) followed by zeroing the memory. Code blocks for visualizing results were removed. Because the syntax of Java and C++ is relatively similar, other Java codes were directly reused in C++. Finally, the converted C++ program was compiled in Visual C++ 2005 (Microsoft), with the moderate optimization for execution speed (/O2).

*Test images used for comparison of computation speed among different GabRat programs (Table 2)*

A dried specimen of the weevil *Pachyrhynchus tobafolius* (Coleoptera: Curculionidae) was obtained from the insect collection of the National Taiwan Normal University. The specimen was photographed using a Nikon D750 digital camera equipped with a 105 mm macro lens (AF-S VR Micro NIKKOR, 105 mm F/2.8G IF-ED). The insect photo was resized so that the body width becomes 500 pixels. For the background image, forest floor under the natural host trees of *P. tobafolius* was photographed using a Nikon D750 digital camera equipped with a 50 mm lens (AF-S NIKKOR 50mm f/1.8G), in Orchid Island, Taiwan. The background image was also resized and trimmed into 3,000 x 3,000 pixels so that the width and height of the image were equivalent to 4.5 cm in the real metrics. The insect image was trimmed so as only to include the major part of the body (i.e. head, prothorax and the elytra) and placed at the center of the background image (Fig. B). As a result, the number of the contour pixels scanned by GabRat programs was 2428 pixels. The composite image was finally converted to a grayscale image, in which the luminescence of each pixel (*L*) is determined as *L* = 0.299*R* + 0.582*G* + 0.114*B*, where *R*, *G*, and *B* represent the pixel intensities of red, green, and blue channels in RGB color space, respectively.

**References**

Troscianko J, Stevens M. Image Calibration and Analysis Toolbox—a free software suite for objectively measuring reflectance, colour and pattern. *Methods Ecol Evol. 2015;***6**: 1320–1331.

**Figures**


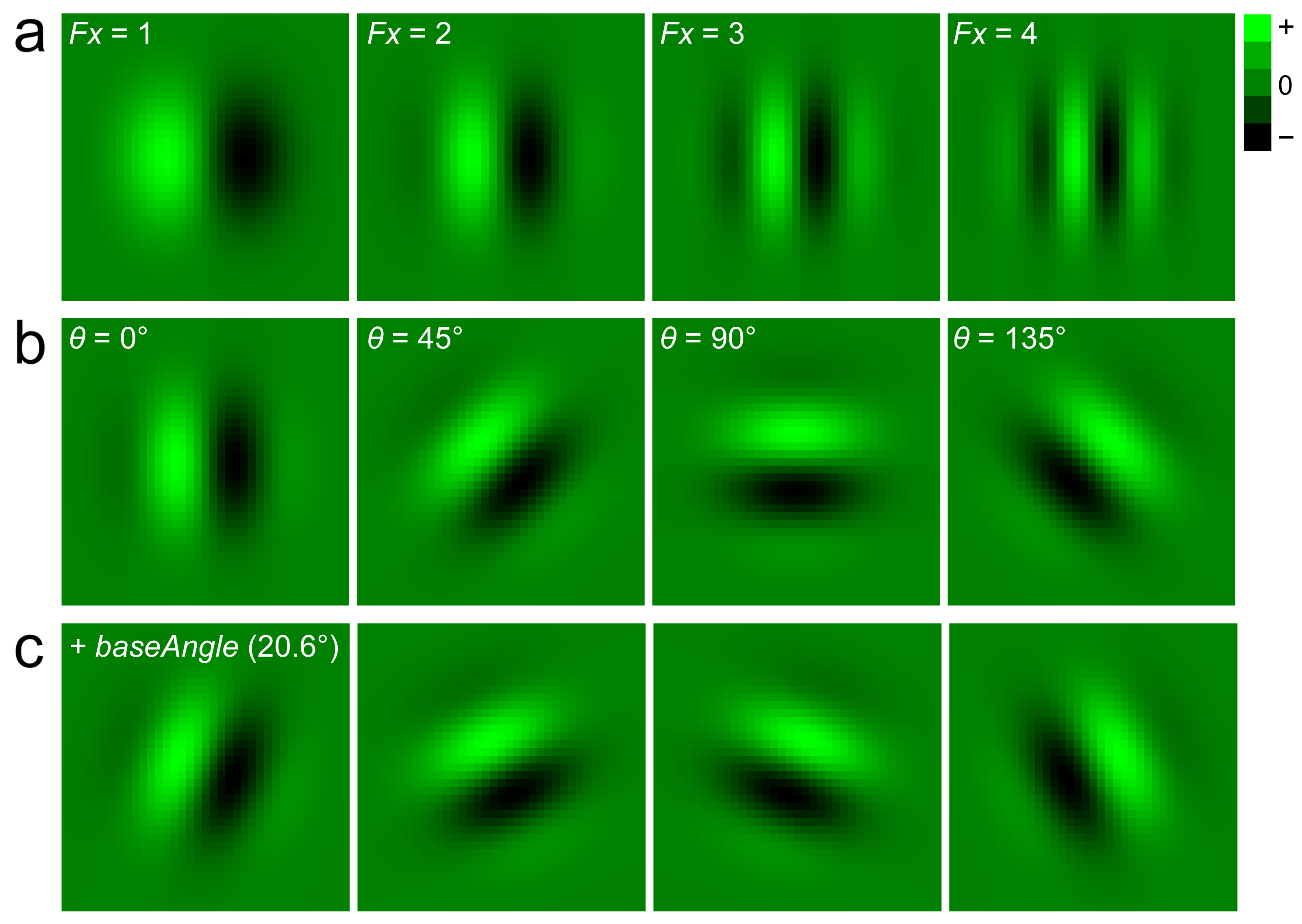


**Fig. A. Variations of Gabor filters with different parameters.** (a) The frequency number, *Fx*, is changed from 1 to 4. (b) The filter angle, *θ*, is changed from 0° to 135° by a step of 45°, which means that *nAngles* parameter is set to 4. *Fx* = 2. (c) Each filter matrix is additionally rotated by a randomly-generated *baseAngle* parameter, which is newly introduced in GabRat-R method. The parameter of the filter size, *σ*, is fixed to 6 in all abovementioned cases, and the actual matrix size (*k* × *k*) is determined as *k* = 6*σ* + 1. The color gradient from black to blight green represents the values of the *k × k* matrix from negative to positive. Note that the values are nearly zero at the center and the peripheral areas of the matrix.


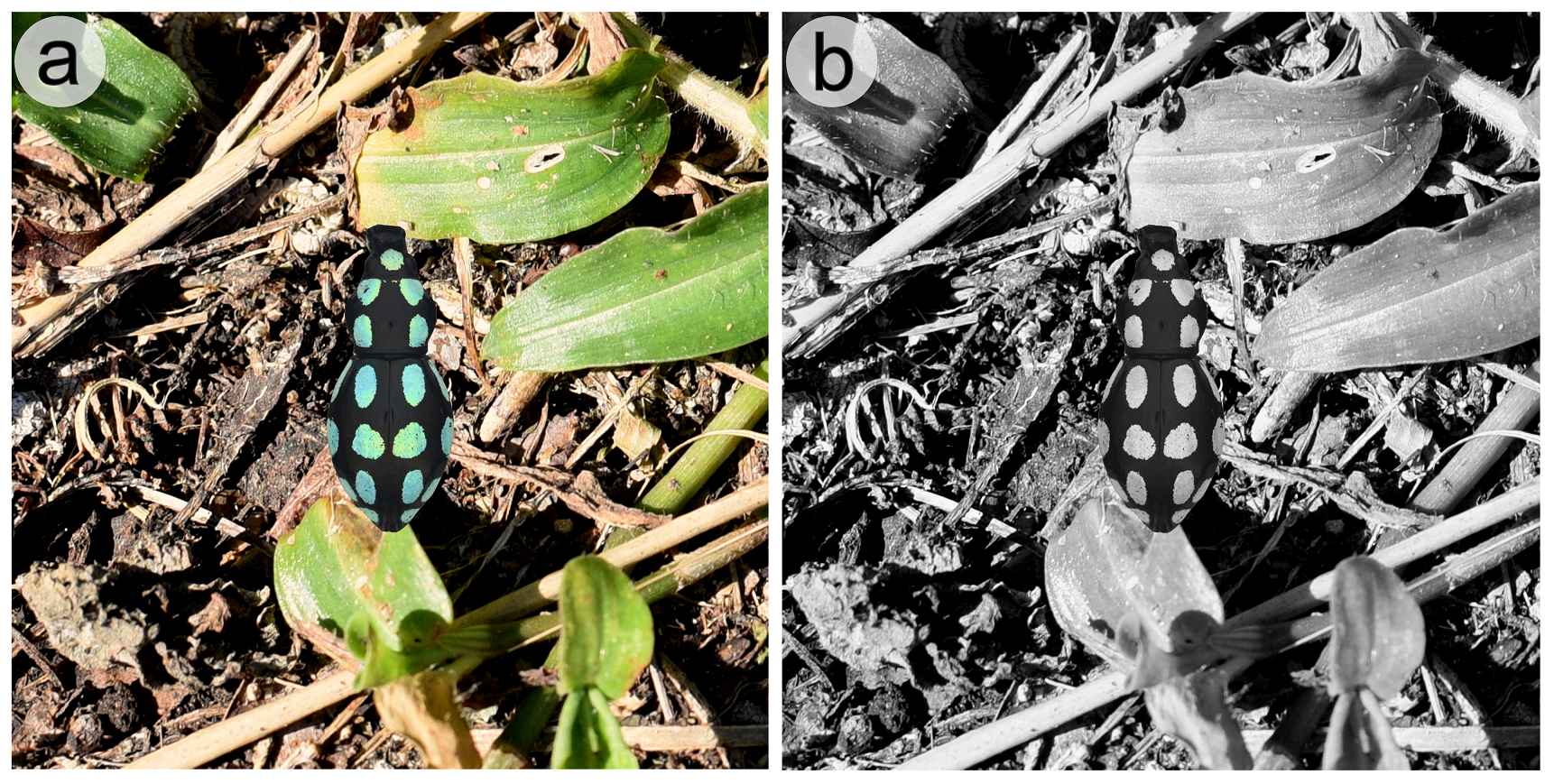


**Fig. B. Images used to test the computational speed of different GabRat programs.** A specimen image of the weevil *Pachyrhynchus tobafolius* was placed on a background image of its natural habitat in Orchid Island, Taiwan. (a) Color image. (b) Grayscale image. The grayscale image was used for the GabRat computations in Table 2 of the main text.

**Codes**

**Code A. C++ header file for the converted GabRat program (GabRat_Disruption_java.h).**

//

// GabRat_Disruption_java.h

//

// Original GabRat algorithm (Troscianko et al. 2017)

// was converted to C++ code by M. Tanahashi (24 Feb 2024)

//

// Original Java code is available at:

// https://github.com/troscianko/micaToolbox

//

#pragma once

#include <my/image/plane.h> // Plane

///////////////////////////////////////

// Store the results

struct GabRatResult

{

int edgeCount;

double gabRatSum;

};

///////////////////////////////////////

// Emulate the Mask class of ImageJ

class MaskJ : public Plane<BYTE>

{

public:

BYTE getPixel(int x, int y) const { if(x<0 || y<0 || x>=Width() || y>=Height()) throw 0; return At(x, y); }

};

///////////////////////////////////////

// Emulate the Image class of ImageJ

class ImageJ : public Plane<float>

{

public:

float getPixelValue(int x, int y) const { if(x<0 || y<0 || x>=Width() || y>=Height()) throw 0; return At(x, y); }

int getWidth() const { return Width(); }

int getHeight() const { return Height(); }

};

///////////////////////////////////

// The Only function to call

GabRatResult GabRatDisduptionOriginal

(

ImageJ& ip,

MaskJ& mask,

int nAngles,

double sigma,

double gamma,

double Fx

);

**Code B. C++ implementation file for the converted GabRat program (GabRat_Disruption_java.cpp).**

//

// GabRat_Disruption_java.cpp

//

// Original GabRat algorithm (Troscianko et al. 2017)

// was converted to C++ code by M. Tanahashi (24 Feb 2024)

//

// Original Java code is available at:

// https://github.com/troscianko/micaToolbox

//

#include "stdafx.h"

#include "GabRat_Disruption_java.h"

#include <math.h> // cos, sin, exp, M_PI

// Internal classes compatible with Java.Math

class Math

{

public:

static double sin(double a) { return ::sin(a); }

static double cos(double a) { return ::cos(a); }

static double exp(double a) { return ::exp(a); }

static double abs(double a) { return ::abs(a); }

static int round(double a) { return(a > 0) ? (int)(a + 0.5) : -(int)(-a + 0.5); }

static double PI;

};

double Math::PI = M_PI;

// The name Rectangle is already defined as global function, thus use JRectangle

struct JRectangle

{

public:

int x;

int y;

int width;

int height;

JRectangle(int x, int y, int width, int height) : x(x), y(y), width(width), height(height) {}

};

// Emulate Java array

template<class T> class JArray

{

protected:

int size;

T* m_pArray;

public:

JArray(int n) { size = n; m_pArray = new T[n]; memset(m_pArray, 0, sizeof(T) * n); }

~JArray() { delete[] m_pArray; m_pArray = NULL; size = 0; }

int Size() const { return size; }

T* Buffer() { return m_pArray; }

const T* Buffer() const { return m_pArray; }

operator T*() { return m_pArray; }

operator const T*() const { return m_pArray; }

#ifdef _DEBUG

void Validate(int i) const { if(i < 0 || i >= size) throw 0; }

T& operator[] (int i) { Validate(i); return *(m_pArray + i); }

const T& operator[] (int i) const { Validate(i); return *(m_pArray + i); }

#else

void Validate(int i) const {}

T& operator[] (int i) { return *(m_pArray + i); }

const T& operator[] (int i) const { return *(m_pArray + i); }

#endif

};

// The Only function to call

GabRatResult GabRatDisduptionOriginal(ImageJ& ip, MaskJ& mask, int nAngles, double sigma, double gamma, double Fx)

{

double psi = Math::PI / 4.0 * 2; // Phase

if(nAngles/2 != Math::round(nAngles/2))

nAngles = nAngles -1;

double sigma_x = sigma;

double sigma_y = sigma / gamma;

double largerSigma = 0.0;

// Decide size of the filters based on the sigma

if(sigma_x > sigma_y)

largerSigma = sigma_x;

else largerSigma = sigma_y;

if(largerSigma < 1)

largerSigma = 1;

double sigma_x2 = sigma_x * sigma_x;

double sigma_y2 = sigma_y * sigma_y;

int filterSizeX = (int) Math::round(6 * largerSigma + 1);

int filterSizeY = (int) Math::round(6 * largerSigma + 1);

JArray<float> kernelArray(nAngles * filterSizeX * filterSizeY);

int middleX = Math::round(filterSizeX / 2) + 1; // Note: It is equivalent to (int)(filterSizeX / 2) + 1 since filterSizeX is integer

int middleY = Math::round(filterSizeY / 2) + 1; // and is NOT the center (ex. Size = 9 -> mid = 5, but true center is mod = 4)

double rotationAngle = Math::PI/nAngles;

double theta = 0.0;

double xPrime = 0.0;

double yPrime = 0.0;

double a = 0.0;

double c = 0.0;

for(int i=0; i<nAngles; i++){

theta = rotationAngle * i;

for(int y=0; y<filterSizeY; y++){

for(int x=0; x<filterSizeX; x++){

xPrime = (x-middleX+1) * Math::cos(theta) + (y-middleY+1) * Math::sin(theta);

yPrime = (y-middleY+1) * Math::cos(theta) - (x-middleX+1) * Math::sin(theta);

a = 1.0 / ( 2.0 * Math::PI * sigma_x * sigma_y ) * Math::exp(-0.5 * (xPrime*xPrime / sigma_x2 + yPrime*yPrime / sigma_y2) );

c = Math::cos( 2.0 * Math::PI * (Fx * xPrime) / filterSizeX + psi);

kernelArray[(i*filterSizeX * filterSizeY)+(y*filterSizeX)+x] = (float) (a*c);

}//x

}//y

}// i (angles)

// --------------------- Get ROI Mask and Outline---------------------------------

JRectangle r(0, 0, ip.getWidth(), ip.getHeight());

int w = ip.getWidth();

int h = ip.getHeight();

JArray<float> edgeArray(w*h);

// Find up-down outline (bottom edge)

for (int y=0; y<r.height-1; y++)

for (int x=0; x<r.width; x++)

if(mask.getPixel(x,y) != 0 && mask.getPixel(x,y+1) == 0)

edgeArray[((y+r.y)*w) + x+r.x] = 1;

// Find down-up outline (top edge)

for (int y=0; y<r.height-1; y++)

for (int x=0; x<r.width; x++)

if(mask.getPixel(x,y+1) != 0 && mask.getPixel(x,y) == 0)

edgeArray[(((y+1)+r.y)*w) + x+r.x] = 1;

// Find left-right outline (right edge)

for (int y=0; y<r.height; y++)

for (int x=0; x<r.width-1; x++)

if(mask.getPixel(x,y) != 0 && mask.getPixel(x+1,y) == 0)

edgeArray[((y+r.y)*w) + x+r.x] = 1;

// Find right-left outline (left edge)

for (int y=0; y<r.height; y++)

for (int x=0; x<r.width-1; x++)

if(mask.getPixel(x+1,y) != 0 && mask.getPixel(x,y) == 0)

edgeArray[((y+r.y)*w) + x+r.x+1] = 1;

// Fill in the mask edges that meet the boundaries

for (int y=0; y<r.height; y++){

if(mask.getPixel(0,y) != 0)

edgeArray[((y+r.y)*w) + r.x] = 1;

if(mask.getPixel(r.width-1,y) != 0)

edgeArray[((y+r.y)*w) + r.width+r.x-1] = 1;

}

for (int x=0; x<r.width; x++){

if(mask.getPixel(x,0) != 0)

edgeArray[(r.y*w) + x+r.x] = 1;

if(mask.getPixel(x,r.height-1) != 0)

edgeArray[((r.height+r.y-1)*w) + x+r.x] = 1;

}

// Create arrays with x & y coordinates of the edge points

int edgeCount = 0;

for(int i=0; i<w*h; i++)

if(edgeArray[i] == 1)

edgeCount++;

int* xCoords = new int[edgeCount];

int* yCoords = new int[edgeCount];

edgeCount = 0;

for(int y=r.y; y<r.height+r.y; y++)

for(int x=r.x; x<r.width+r.x; x++)

if(edgeArray[(y*w)+x] == 1){

xCoords[edgeCount] = x;

yCoords[edgeCount] = y;

edgeCount++;

}

// Create mask image with full size for Gabor convolution

JArray<float> maskArray(w*h);

for (int y=0; y<r.height; y++)

for (int x=0; x<r.width; x++)

if(mask.getPixel(x,y) != 0)

maskArray[((y+r.y)*w) + x+r.x] = 1;

// ----------------------------------------CONVOLVE-----------------------------------------

JArray<double> maskGaborData(edgeCount * nAngles);

JArray<double> imageGaborData(edgeCount * nAngles);

int xFocus = 0;

int yFocus = 0;

for(int i=0; i<nAngles; i++){

for(int j=0; j<edgeCount; j++){

xFocus = xCoords[j]-middleX+1; // I tested this centre, and it's correct

yFocus = yCoords[j]-middleY+1;

for(int y=0; y<filterSizeY; y++)

if(yFocus+y < h && yFocus+y > 0)

for(int x=0; x<filterSizeX; x++)

if(xFocus+x < w && xFocus+x > 0){

//work out the angle of the object outline at each point on the edge

maskGaborData[(i*edgeCount)+j] = maskGaborData[(i*edgeCount)+j] + (maskArray[((yFocus+y)*w)+(xFocus+x)] * kernelArray[(i*filterSizeX * filterSizeY)+(y*filterSizeX)+x]);

// parallel to the outline and orthogonal to the outline

imageGaborData[(i*edgeCount)+j] = imageGaborData[(i*edgeCount)+j] + (ip.getPixelValue(xFocus+x, yFocus+y) * kernelArray[(i*filterSizeX * filterSizeY)+(y*filterSizeX)+x]);

}//x

}//j

}// i (angles)

// --------------------CONVERT TO ABSOLUTE-------------------------

for(int i=0; i<nAngles*edgeCount; i++){

maskGaborData[i] = Math::abs(maskGaborData[i]);

imageGaborData[i] = Math::abs(imageGaborData[i]);

}

// --------------FIND ANGLE OF THE TARGET'S EDGE-----------------------

JArray<int> edgeAngle(edgeCount);

JArray<double> maskEnergy(edgeCount);

double energy = 0.0;

for(int j=0; j<edgeCount; j++)

for(int i=0; i<nAngles; i++){

energy = maskGaborData[(i*edgeCount)+j];

if(energy > maskEnergy[j]){

edgeAngle[j] = i;

maskEnergy[j] = energy;

}

}

//-------------CALCULATE GabRat EDGE DISRUPTION RATIO----------------------

JArray<double> gabRat(edgeCount);

JArray<double> gabRatE(edgeCount);

JArray<double> orthEnergy(edgeCount);

JArray<double> paraEnergy(edgeCount);

int paraAngle = 0;

int orthAngle = 0;

for(int j=0; j<edgeCount; j++){

paraAngle = edgeAngle[j];

// Calculate opposite (orthogonal) angle to edge

orthAngle = paraAngle - (nAngles/2);

if(paraAngle < nAngles/2)

orthAngle = paraAngle + (nAngles/2);

paraEnergy[j] = imageGaborData[(paraAngle*edgeCount)+j];

orthEnergy[j] = imageGaborData[(orthAngle*edgeCount)+j];

gabRat[j] = orthEnergy[j] / ( orthEnergy[j] + paraEnergy[j] );

gabRatE[j] = (orthEnergy[j] / ( orthEnergy[j] + paraEnergy[j] )) * orthEnergy[j];

}

double gabRatSum = 0.0;

double gabRatESum = 0.0;

for(int j=0; j<edgeCount; j++){

gabRatSum += gabRat[j];

gabRatESum += gabRatE[j];

}

// Result

GabRatResult gabrat;

gabrat.edgeCount = edgeCount;

gabrat.gabRatSum = gabRatSum;

return gabrat;

}
